# Effects of Ocean Acidification on Learning in Coral Reef Fishes

**DOI:** 10.1371/journal.pone.0031478

**Published:** 2012-02-06

**Authors:** Maud C. O. Ferrari, Rachel P. Manassa, Danielle L. Dixson, Philip L. Munday, Mark I. McCormick, Mark G. Meekan, Andrew Sih, Douglas P. Chivers

**Affiliations:** 1 Department of Environmental Science and Policy, University of California Davis, Davis, California, United States of America; 2 ARC Centre of Excellence for Coral Reef Studies, and School of Marine and Tropical Biology, James Cook University, Townsville, Queensland, Australia; 3 Australian Institute of Marine Science, UWA Ocean Sciences Centre (MO96), Crawley, Western Australia, Australia; 4 Department of Biology, University of Saskatchewan, Saskatoon, Saskatchewan, Canada; Institute of Marine Research, Norway

## Abstract

Ocean acidification has the potential to cause dramatic changes in marine ecosystems. Larval damselfish exposed to concentrations of CO_2_ predicted to occur in the mid- to late-century show maladaptive responses to predator cues. However, there is considerable variation both within and between species in CO_2_ effects, whereby some individuals are unaffected at particular CO_2_ concentrations while others show maladaptive responses to predator odour. Our goal was to test whether learning via chemical or visual information would be impaired by ocean acidification and ultimately, whether learning can mitigate the effects of ocean acidification by restoring the appropriate responses of prey to predators. Using two highly efficient and widespread mechanisms for predator learning, we compared the behaviour of pre-settlement damselfish *Pomacentrus amboinensis* that were exposed to 440 µatm CO_2_ (current day levels) or 850 µatm CO_2_, a concentration predicted to occur in the ocean before the end of this century. We found that, regardless of the method of learning, damselfish exposed to elevated CO_2_ failed to learn to respond appropriately to a common predator, the dottyback, *Pseudochromis fuscus*. To determine whether the lack of response was due to a failure in learning or rather a short-term shift in trade-offs preventing the fish from displaying overt antipredator responses, we conditioned 440 or 700 µatm-CO_2_ fish to learn to recognize a dottyback as a predator using injured conspecific cues, as in Experiment 1. When tested one day post-conditioning, CO_2_ exposed fish failed to respond to predator odour. When tested 5 days post-conditioning, CO_2_ exposed fish still failed to show an antipredator response to the dottyback odour, despite the fact that both control and CO_2_-treated fish responded to a general risk cue (injured conspecific cues). These results indicate that exposure to CO_2_ may alter the cognitive ability of juvenile fish and render learning ineffective.

## Introduction

Ocean acidification, caused by the uptake of additional carbon dioxide (CO_2_) from the atmosphere, is a significant threat to marine ecosystems [1], [2], [3], [4]. A rapid increase in CO_2_ in the atmosphere leads to a lowering of the pH of the oceans, as additional CO_2_ reacts with water to release bicarbonate (HCO_3_^−^) and hydrogen ions (H^+^). This process has resulted in a drop in oceanic pH by 0.1 pH units since pre-industrial times [5] and a further 0.3–04 pH units decrease is predicted by 2100 if current CO_2_ emissions trajectories are maintained [4]. Such a decrease is not novel per se, as geologic records indicate similar situations have occurred in the past, such as during the Paleocene-Eocene period some 56 million years ago [6], [7]. A key question is how biological life will cope with this rapid change in ocean chemistry [5].

The potential effects of acidification on calcifying organisms, such as corals and invertebrates with calcareous exoskeletons, due to the reduced saturation of carbonate ions in the ocean at lower pH [3], [5], [8] is now well-recognised. Much less is known on the consequences of ocean acidification on non-calcifying marine species, such as fish [9], [10]. Indeed, a recent meta-analysis [10] shows that only 25% of the 198 tests reporting ocean acidification effects were performed on non-calcifiers, with only 2% of the studies being done on fishes (the other 23% focusing on algae and aquatic plants). Although early research indicated that very high levels of CO_2_ (>10,000 ppm) were lethal for a number of fish species [11], some fish species appear to be tolerant of mild increases in pCO_2_ [12], [13]. However, non-lethal CO_2_ levels predicted by the end of the century (up to ∼1000 ppm depending on the IPCC scenario chosen) [14] may still lead to negative consequences. For example, Dixson et al. [15] reported that the coral reef clownfish *Amphiprion percula* was affected by CO_2_ exposure so that larvae exposed to CO_2_ levels of 1000 ppm were not able to respond appropriately to the odour of predatory fishes (the rockcod, *Cephalopholis cyanostigma* and a dottyback, *Pseudochromis fuscu*s). Munday et al. [16] provided the first evidence of the fitness costs associated with such effects of CO_2_. Young juveniles of the damselfish, *Pomacentrus wardi*, that were exposed to 850 µatm of CO_2_ and released in the wild suffered an 8-fold increase in predation-related mortality in the first 30 h of settlement, compared to control fish exposed to present-day levels of CO_2_ (440 µatm CO_2_). These results do not reflect a lack of detection of the cues by the fish, as both Dixson et al [15] and Munday et al. [16] showed that juveniles from controls avoided predator odours, while CO_2_-treated juveniles were *attracted to* predator odours.

Recent studies have shown a surprising amount of intra- and inter-specific variation in the effects of CO_2_ on fishes [16], [17]. At levels nearing 700 µatm, some individuals consistently display an appropriate response while others consistently show maladaptive responses to predators. Thus, there should exist a time in the future where affected fish (those that do not respond appropriately to predators) will co-occur with unaffected individuals. This could either result in strong directional selection, whereby affected individuals will be removed from the population, or it could delay the effects of CO_2_, by allowing these fish to learn to display the appropriate response by copying the behaviour of non-affected individuals. Thus, the extent to which appropriate responses to predators may be acquired is a key question. If this is possible, it might mitigate the effects of ocean acidification on predator-prey interactions.

Some coral reef fishes do not show innate recognition of predators [18]. Learning is key to acquire new knowledge, skills and behaviours, and interaction and experience with predators are among the most efficient means of learning the identity of predators, due to the immediate costs (i.e., injury or death) associated with a lack of an appropriate response by potential prey. For aquatic species, one way to learn to recognize predators is through the simultaneous detection of novel predators and cues from injured conspecifics (reviewed by [19]). Cues from injured conspecifics (or ‘alarm cues’) are known to elicit immediate and dramatic antipredator responses, due to the highly reliable nature of those cues in a predation context; they are only released through mechanical damage to the skin of the prey, typically during a predator attack. Such learning is highly efficient – one-time learning – and widespread, from flatworms to larval amphibians [19]. The first goal of our study was thus to test if 850 µatm CO_2_-exposed fish would acquire recognition of novel predators through this learning process.

Another form of antipredator learning involves social learning, whereby naive individuals learn by observing more experienced conspecifics respond to a predator ([20], [21] for reviews). Social learning may be particularly important for coral reef fishes, as they often colonize corals at high densities and have opportunities to observe the behaviours of resident conspecifics and heterospecifics. If individuals that are affected by CO_2_ can learn to recognize predators from unaffected individuals, then the negative effects of CO_2_ exposure may be reduced. Thus, the goal of a second experiment was to investigate how exposure to elevated CO_2_ affected the ability to acquire recognition of a novel predator from individuals not affected by CO_2_.

A failure to respond to predator cues following a conditioning event may be explained one of two ways: 1) the prey may have failed to learn the predator as a danger, or 2) the prey successfully learned to recognize the predator, but intrinsic factors may prevent them from showing an overt antipredator response to the cues at the time of testing. One such intrinsic factor is hunger level. For instance, Brown et al. [22] showed that hungry fathead minnows, *Pimephales promelas*, still learned to recognize pike, *Esox lucius*, as a predator despite the absence of an alarm response during conditioning. When subsequently fed, the minnows displayed antipredator responses similar to those of well-fed minnows when exposed to pike odour. These results are explained by a shift in foraging trade-offs whereby the need of prey to forage overrides the behavioural responses to the predator. In our situation, it is possible that CO_2_ may alter physiological and foraging needs via another state-dependent factor explaining the lack of response of prey to predator cues. To discriminate between these two options, we performed a third experiment whereby 700-µatm CO_2_-exposed fish that had been conditioned to recognize a predator, via conditioning with injured conspecific cues, were tested for their response to the predator at one day or five days post conditioning. We chose 5 days as previous studies have shown that the CO_2_ effects only last up to four days after the fish have been returned to control water [16].

Our study examined these questions in the context of a coral reef ecosystem on the Great Barrier Reef, Australia. Most coral reef fishes have a pelagic larval stage that resides in the plankton for a period of weeks to months [23]. At the end of this phase, juvenile fish must locate suitable benthic habitat and in doing so, face a new and abundant array of predatory reef fishes. Predators may remove up to 60% of newly settling fish in a single night [24], creating population bottlenecks. In the days immediately prior to settlement, juvenile fish can be captured away from the reef in large numbers using light traps [25], [26]. Although they have juvenile form and colouration, these individuals are naïve to the suite of predators that await them on the reef. Learning to recognize predators upon settlement is a critical step in the life history of these fish. Our system provides a unique opportunity to examine interactions between learning behaviour, predation and the effects of ocean acidification.

## Methods

### Test subjects and CO_2_ treatment

Experiments took place at the Lizard Island Research Station (14°40′S, 145°28′E), on the Great Barrier Reef, Australia, in November and December 2009 (experiments 1 and 2) and 2010 (experiment 3). We used established protocols to capture and treat our fish [16], [27]. Pre-settlement juveniles (16–21 days old) of *Pomacentrus amboinensis* were caught overnight in light traps [26] moored >100 m off the reef at Lizard Island. Light traps collect these fish immediately prior to their arrival on the reef at the end of the planktonic larval stage [28]. Every morning, *P. amboinensis* collected in the traps were transferred to 35-L rearing aquariums at 440 (present-day control CO_2_ levels), 700 or 850 µatm CO_2_. Pomacentrid larvae exposed to elevated CO_2_ over a few days showed identical behavioural impairments as larvae raised under the same CO_2_ levels from birth [16], indicating that the alteration in behaviour were not due to a sudden CO_2_ exposure. Moreover, given their bipartite life history, juvenile damselfish would naturally be exposed to a change in CO_2_ conditions, when they recruit from the open ocean, where CO_2_ conditions are relatively stable, to the coral reef where pCO_2_ can fluctuate significantly on a daily basis due the net effects of photosynthesis, respiration and calcification [29], [30].

CO_2_ treatments were maintained by CO_2_ dosing to a set pH_NBS_ following standard techniques for ocean acidification research, as set out in the Best Practices Guides for Ocean Acidification Research [31]. Seawater was pumped from the ocean into 4×60 L sumps where it was diffused with ambient air (control) or CO_2_ to achieve a pH of approximately 8.15 (control), 7.97 or 7.89. The reduced pH values were selected to achieve the approximate CO_2_ conditions required, based on preliminary observations of total alkalinity, salinity and temperature of seawater at Lizard Island. A pH-controller (Tunze Aquarientechnik, Penzberg, Germany) was attached to each of the CO_2_ treated sumps to maintain pH at the desired level. A solenoid injected a slow stream of CO_2_ into a powerhead at the bottom of the sump whenever the pH of the seawater rose above the set point. The powerhead rapidly dissolved CO_2_ into the seawater and also served as a vigorous stirrer. Equilibrated seawater from each sump was supplied at a rate of ∼500 ml.min^−1^ to four replicate 35-L aquariums, each housing a group of larval fishes. To maintain oxygen levels and the required *p*CO2 levels, aquariums were individually aerated with air (control ∼440 µatm) or CO_2_-enriched air (∼700, or 850 µatm). The concentration of CO_2_-enriched air was controlled by a scientific-grade pressure regulator and precision needle valve and measured continuously with an infrared CO_2_ probe (Vaisala GM70, Vaisala, Helsinki, Finland). Temperature and pH_NBS_ of each aquarium was measured each morning and afternoon using an HQ40d pH meter (Hach, Loveland, Colorado, USA) calibrated with fresh buffers. Total alkalinity of seawater was estimated by Gran titration from water samples taken twice weekly from each CO_2_ treatment. Alkalinity standardizations performed before processing each batch achieved accuracy within 1% of certified reference material from Dr. A. Dickson (Scripps Oceanographic Institute). Average seawater *p*CO2 was calculated using these parameters in the program CO2SYS and using the constants of Mehrbach et al. [32] refit by Dickson & Millero [33]. Estimated seawater parameters are shown in Table 1.

**Table 1:** Mean (± SD) seawater parameters in the experimental system.

pH_NBS_	Temp °C	Salinity ppt	TA (µmol.kg^−1^SW)	*p*CO_2_
8.15 (0.04)	27.66 (0.98)	35	2269.66 (15.01)	440.53 (44.46)
7.97 (0.06)	27.59 (0.97)	35	2259.87 (11.55)	718.37 (110.82)
7.89 (0.06)	27.74 (0.99)	35	2261.23 (14.92)	879.95 (140.64)

Temperature, pH salinity, and total alkalinity (TA) were measured directly. *p*CO_2_ was estimated from these parameters using CO2SYS.

Young damselfishes were fed freshly hatched *Artemia nauplii* three times a day. The fish were treated for 4 consecutive days and then used in our experiment immediately after the treatment period was over. Due to experimental limitations in the amount of CO_2_ water that could be produced daily, it was not possible to test CO_2_-treated fish in CO_2_-enriched water. Thus, the experimental manipulations described thereafter took place in control water. This methodology was successfully used previously [16], [17], [34]. Juvenile damselfish have also been shown to display the same behavioural alteration in CO_2_-enriched as in control water after a 4-day CO_2_ exposure period [16]. Fish treated with 700–850 µatm CO_2_ retain their CO_2_-induced impaired behavioural responses for at least 48 h after being transferred back into control water, but no longer than 4 days [16].

### Experiment 1: Acquired predator recognition via pairing with cues from injured conspecifics

Our first experiment investigated the ability of CO_2_-treated fish to respond to predator odour following conditioning with cues from injured conspecifics. The learning procedure is a 2-step process that first involves a conditioning phase where fish are exposed to cues of injured conspecifics paired with those of a novel predator and second, a testing phase, where fish are exposed to the predator cue alone to measure any learned antipredator response. Our experimental set-up followed a complete 2×2×2 design, consisting of conditioning either control or 850 µatm CO_2_-treated fish with the odour of a predatory dottyback, a common predator of newly-settled damselfishes at Lizard Island [35], paired with either water (pseudo-conditioning) or cues from injured conspecifics (true conditioning). Later, the fish were tested for their response to either the dottyback odour or a water control. The group of individuals that were pseudo-conditioned should not have acquired recognition of the predator, while predator odour should be recognised as a risky stimulus by the group that were exposed to the cues from injured conspecifics.

#### Conditioning phase

At least 6 h prior to conditioning, larvae were removed from their respective CO_2_ treatment, and placed individually in 20-L flow-through tanks (32×16×16 cm) equipped with sand, a small piece of dead coral as a shelter, an airstone, and a 1.5 m long injection tube used to introduce stimuli into the tank. Each tank was covered on three sides with black plastic to avoid visual transfer of information from surrounding tanks. In addition, a black plastic curtain was hung in front of the tanks to minimize disturbance to the fish by the movement of the observer. One h after adding fish to the conditioning tanks and again, 1 h prior to conditioning, the fish were fed *ad lib* with *Artemia* larvae. Water flow was turned off 30 min prior to conditioning the fish. In half of the tanks, we introduced 5 mL of injured conspecific cues paired with 20 mL of dottyback odour, while the other half received 5 mL of seawater paired with 20 mL of dottyback odour. The concentrations we used are based on previously published studies [17], [18]. After 1 h, we turned the flow-through system back on, transferred the fish into their testing tanks with the water flow on, and fed them ad lib 30 min later.

Cues from injured conspecifics were prepared fresh, by gently slicing the side of a sacrificed individual (JCU Animal Ethics Protocol A1067) and rinsing it with fresh seawater. A preliminary experiment showed that cues produced by making 4 cuts on each side of a fish were enough to elicit an overt antipredator response in juvenile damselfish when injected into the tanks. Thus, to minimize the number of fish sacrificed, we made 12 cuts on each side of a fish and rinsed it with 15 mL of seawater in a glass Petri dish to obtain enough cues for 3 conditioning events. We repeated this procedure until we had enough cues to condition all the tanks for that day, and mixed all the cues together prior to injection. All cues were used within 15 min of being made to ensure their potency [18]. Dottyback were collected 3 weeks prior to our experiment while diving in the lagoon at Lizard Island using hand nets and anaesthetic clove oil mixed with alcohol and seawater. Two yellow morph dottybacks (6.5 and 7.1 cm standard length) were maintained in a 70-L tank of aerated water where 60% of the water was changed daily. The dottyback were fed prior to the water change with INVE Aquaculture Nutrition 12/20 pellets. Water taken from the dottyback tank was used as our predator odour and was injected into our experimental tank within 20 min of being collected.

#### Testing phase

Trials began between 4 and 8 hours after transfer of juvenile fish into the testing tank. Test and conditioning tanks were identical, with the exception that a 4×4 cm grid was drawn on the side of the test tank to help the observer record positions of the fish during the experiment. One h prior to testing, the juvenile fish were fed and the flow-through system was tuned off 30 min later. Behavioural observations of the fish were conducted during this phase. The order of treatments was randomized.

#### Behavioural bioassay

To stimulate activity, we injected small quantities of food into the tank, on the opposite side of the coral shelter, creating a choice for juveniles to either forage or take refuge within the coral head. During each observation period, we measured 3 behaviours: (1) the total number of feeding strikes displayed by the fish, regardless of whether they were successful at capturing a food item or not; (2) the total number of lines the fish crossed during the observation period, using the 4×4-cm grid drawn on the side of the tank. A line was counted as crossed when the entire body of the fish crossed a line. This behaviour represents a measure of the swimming activity of the fish; (3) the total number of different squares visited during the observation period. This represented the 2-dimensional area of activity of the fish, and is a standard technique used to measure activity [18]. Prey fishes exposed to risk typically decrease or stop feeding, decrease their swimming activity and reduce their area of activity [36], [37].

Initially, the juvenile fish were fed 2.5 mL of food (seawater containing ∼250 *Artemia* larvae.mL^−1^) to remove the possibility of a “feeding frenzy” effect at the start of the bioassay. Pre-stimulus observation began 5 min later, when another 2.5 mL of food was injected into the tank. At the end of this 5-min pre-stimulus observation period, 20 ml of dottyback odour or 20 mL of water were introduced into the tank followed by 2.5 mL of food. The behaviour of the juvenile was then observed for 5 min. The experimenter was blind to the treatment during the observation. To control for any day effect, we tested the same number of fish from each of the treatment groups each day. We ran 16–17 replicates in each of the 8 treatment groups, testing a total of 129 fish.

### Experiment 2: Acquired predator recognition via visual cues from conspecifics

This experiment was designed to test whether the fish could acquire recognition of predators via visual cues from conspecifics, and in particular, whether this ability was impaired by exposure to 850 µatm CO_2_ concentrations. Similar to Experiment 1, the conditioning with odour cues from injured conspecifics and social learning procedures are divided into two phases: the conditioning phase consisting of pairing predator-naive (hereafter ‘learner’) and predator-experienced (hereafter ‘tutor’) individuals and exposing them to predator cues. At this time, the naive learner individual has an opportunity to observe the behavioural response displayed by the experienced tutor toward the predator cues and thus acquire recognition of the cues as a potential threat. In the testing phase, the tutor is removed and the naïve fish subjected to the predator cues. Our experimental design followed a 2×2×2 design, consisting of pairing a naive learner raised under normal or 850 µatm CO_2_ (CO_2_ effect on learner) to a tutor that was either naive or experienced with dottybacks (tutor experience), and then exposing the pair to dottyback odour. During the testing phase, the observers were exposed to water or dottyback odour (testing cue) and their antipredator responses were measured. We predicted that learner fish paired with naive tutors would not learn to recognize the predator as threatening, and that learning to recognize predators from tutors would potentially be reduced if learners were exposed to high CO_2_ concentrations.

#### Naive and experienced tutors

Presettlement juvenile *P. amboinensis* were collected from the light traps in the morning and conditioned to be used as tutors the following day. To distinguish the tutors from learner fish, we marked the tutors with a small colored elastomer tag injected under the skin on their dorsal side behind their dorsal fin. This tagging does not influence the behaviour or survivorship of juvenile damselfishes [38]. Tutors were then randomly placed in conditioning tanks identical to those described in the previous experiment, and underwent a conditioning identical to the one described for Experiment 1. Half of the tutors were conditioned via pairing of injured conspecific cues and dottyback odour, hence rendering them ‘experienced’ to the dottyback predator, while the other half received dottyback odour paired with water (pseudo-conditioning), which kept them ‘naive’ with regards to the dottyback.

#### Conditioning phase

In a flow-through conditioning tank, we paired one naive or one experienced tutor with a learner fish that was raised for 4 days under normal or 850 µatm CO_2_ levels. To control for day effects, we conditioned and tested the same number of each of the four pairing combination each day. Thirty min after pairing them, the fish were fed to satiation. The next morning, the fish were fed again. One h after feeding, the flow-through system was turned off and the conditioning phase began 20 min later. To ensure an overt antipredator response from the tutor fish, we injected 5 mL of *Artemia* in the tank 5 min prior to conditioning. We then injected 2.5 mL of *Artemia*, followed by 20 mL of dottyback odour. We left the fish undisturbed for 1 h, then turned the water flow back on and removed the tutor fish.

#### Testing phase

This phase took place between 4 and 8 h following the conditioning phase. The experimental setup, behavioural bioassay and methodology and cues were identical to the ones described for Experiment 1. The fish were tested for a response to 20 mL of seawater or 20 mL of dottyback odour. We ran 16 replicates in each of the 8 treatment groups, testing a total of 128 fish. The order of testing was randomized among treatments.

### Experiment 3: Is CO_2_ exposure inducing a lack of learning or simply a lack of response?

This experiment was designed to test whether fish that did not display an antipredator response after being conditioned in elevated-CO_2_ water, would subsequently respond to the predator once the CO_2_ effects wore off. Juvenile damselfish exposed to control or 700 µatm CO_2_-levels were conditioned via injured conspecific cues to recognize a predatory dottyback following the same methodology as Experiment 1. All fish were exposed to 5 mL of injured conspecific cues paired with 20 mL of dottyback odour. Although the goal of the experiment was to test for residual CO_2_ effects post-CO_2_ treatment, we needed to ascertain that the results observed in Experiment 1 with 850 µatm CO_2_ fish were also observable with 700 µatm CO_2_ fish. Thus, as in Experiment 1, a group fish was tested for their behavioural response to the predator odour or a water control one day post-conditioning. The rest of the fish were tested 5 days post-conditioning for their response to the predator odour, a water control or an injured conspecific cue control. The water served as a negative control, while the injured conspecific cues served as a positive control, as they elicit overt antipredator responses independently of experience. Hence, we predicted that if fish are able to display an overt antipredator response to injured conspecific cues, they should also be able to display an antipredator response when exposed to the predator odour, assuming they have successfully learned to recognize the odour as a risky stimulus during the conditioning phase. Conditioning and testing protocols were identical to those described in Experiment 1. We conditioned a total of 75 fish.

### Statistical analysis

For all experiments and all variables, no pre-stimulus difference was found among treatments. Thus, we used the raw data to compute change in activity from the pre-stimulus baseline (post minus pre) for each of the three behaviours. Due to the inter-dependency of the three behaviours, we analyze them together using a multivariate approach (MANOVA). In cases where the data did not meet parametric assumptions, the data were rank-transformed and a non-parametric ANOVA approach (extension of the Kruskall-Wallis test) was used on the transformed data [39]. For Experiment 1, we performed a 3-way MANOVA testing the effect of conditioning, CO_2_ and testing cue on the behaviour of the fish. Due to a significant 3-way interaction, we performed 2-way MANOVAs on each conditioning type (pseudo-conditioning with water and true conditioning with injured cues) independently, to investigate the effect of CO_2_ (control vs 850 µatm) and testing cue (water vs predator odour) on the responses of fish. Similarly, for Experiment 2, we performed a 3-way MANOVA, followed by 2-way MANOVAs on each tutor type (naive and experienced tutors) independently, to investigate the effects of CO_2_ (control vs. 850 µatm) and testing cue on the responses of the fish. For experiment 3, we first established that 700 µatm CO_2_-treated fish did not learn to recognize the predator by conducting a 2-way ANOVA, testing the effect of CO_2_ treatment (control vs 700 µatm) and testing cue (water vs predator odour) on the antipredator response of the fish one day post-conditioning. We then conducted a 2-way ANOVA, testing the effect of CO_2_ (control vs 700 µatm) and testing cue (water vs predator odour vs injured conspecific cues) on the response of the fish 5 days post conditioning.

## Results

### Experiment 1

**Figure 1:**
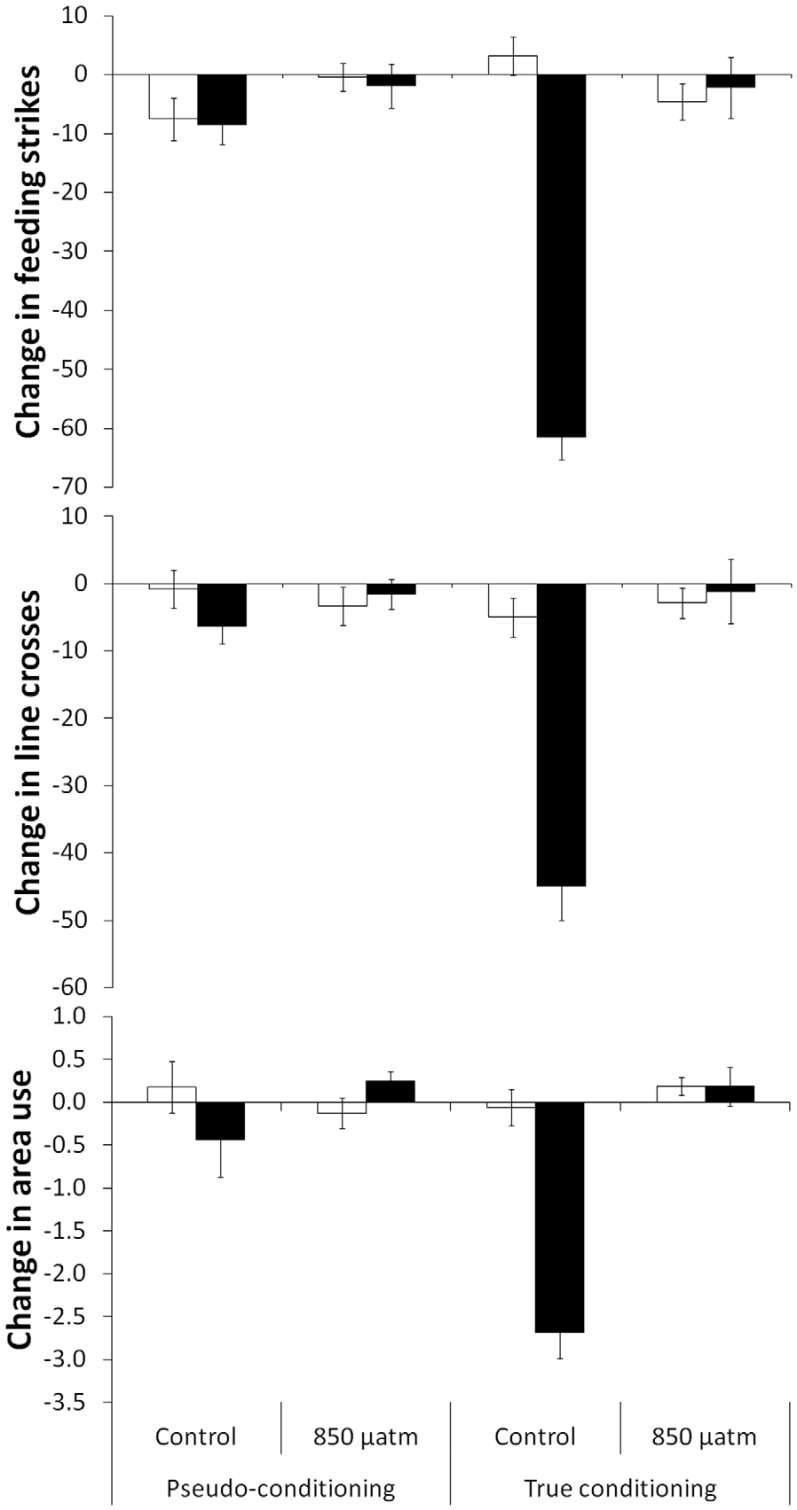
Mean change in number of feeding strikes (top), line crosses (middle) and area use (bottom) from the pre-stimulus period for fish exposed to water (empty bars) or predator odour (solid bars). Fish were either raised under current-level CO_2_ (control) or elevated CO_2_ (850 µatm) and conditioned by pairing predator odour paired with either alarm cues (true conditioning) or water (pseudo-conditioning). (N = 16/treatment).

The antipredator responses displayed by the fish were affected by the cues to which they were exposed, the conditioning they undertook and the CO_2_ levels at which they were maintained (3-way non-parametric MANOVA: Pillai's Trace: Cue×CO_2_×Conditioning: H_3,119_ = 5.2, P = 0.002, Figure 1). The responses of fish pseudo-conditioned with water were affected by neither CO_2_ nor cue (2-way MANOVA: Pillai's Trace: CO_2_: H_3,59_ = 0.8, P>0.4, Cue: H_3,59_ = 0.4, P>0.7; CO_2_×Cue: H_3,59_ = 1.8, P = 0.16). However, the responses of fish that were conditioned to recognize the predator with injured conspecific cues (true conditioning) was dependent on both CO_2_ and cue (CO_2_×Cue: H_3,58_ = 16.3, P<0.001). More specifically, CO_2_ did not affect the responses of fish to water (F_3,28_ = 1.1, P>0.3), but rather that to predator odour (H_3,28_ = 29.5, P<0.001). In addition, fish exposed to 850 µatm CO_2_ did not respond differently to water and predator odour (F_3,28_ = 0.01, P>0.9).

### Experiment 2

**Figure 2:**
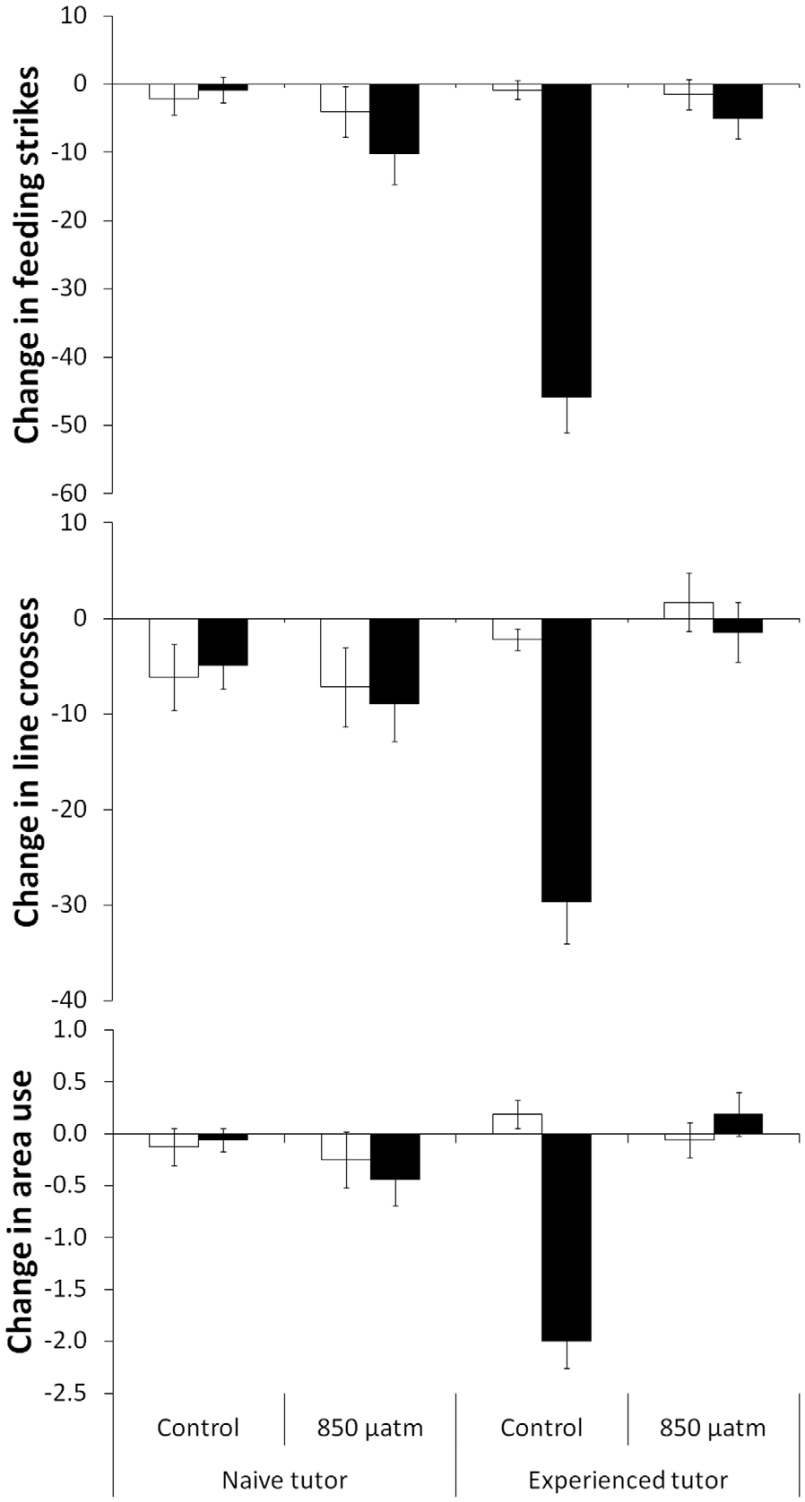
Mean change in number of feeding strikes (top), line crosses (middle) and area use (bottom) from the pre-stimulus period for fish exposed to water (empty bars) or predator odour (solid bars). Fish were either raised under current-level CO_2_ (control) or elevated CO_2_ (850 µatm) and conditioned by being paired with either naive or experienced tutors and exposed to predator odour (N = 16−17/treatment).

The antipredator responses displayed by the fish were affected by the cues to which they were exposed, the experience of their tutor and the CO_2_ levels at which they were maintained (3-way non-parametric MANOVA: Pillai's Trace: Cue×CO_2_×Conditioning: H_3,118_ = 9.7, P<0.001, Figure 2). The responses of fish conditioned with naive tutors were affected by neither CO_2_ nor cue (2-way MANOVA: Pillai's Trace: CO_2_: H_3,58_ = 1.0, P>0.4, Cue: H_3,58_ = 0.2, P>0.9; CO_2_×Cue: H_3,58_ = 0.6, P>0.6). However, the responses of fish that were conditioned to recognize the predator with alarm cues (true conditioning) was dependent on both CO_2_ and cue (CO_2_×Cue: H_3,58_ = 16.7, P<0.001). More specifically, CO_2_ did not affect the responses of fish to water (F_3,28_ = 1.2, P>0.3), but rather that to predator odour (H_3,28_ = 50.6, P<0.001). In addition, fish exposed to 850 µatm CO_2_ did not respond differently to water and predator odour (F_3,28_ = 0.3, P>0.7).

### Experiment 3

#### Test at Day 1

**Figure 3:**
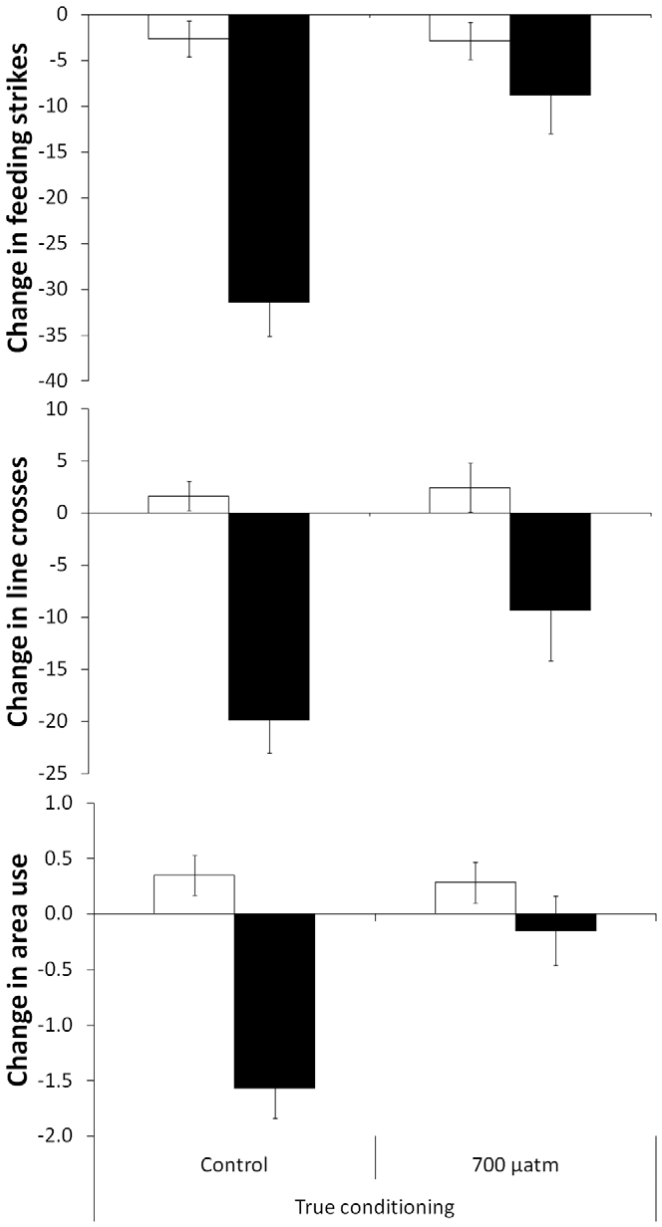
Mean change in number of feeding strikes (top), line crosses (middle) and area use (bottom) from the pre-stimulus period for fish conditioned to recognize a predator and then exposed to water (empty bars) or predator odour (solid bars) after one day. Fish were either raised under current-level CO_2_ (control) or elevated CO_2_ (700 µatm) (N = 20−23/treatment).

Changes in antipredator response were influenced both by CO_2_ and cue (2×2 non-parametric MANOVA, Pillai's Trace: H_3,78_ = 5.7, P = 0.001, Figure 3). The responses of fish to water was not affected by CO_2_ (non-parametric MANOVA, Pillai's Trace: H_3,37_ = 0.1, P>0.9), but their responses to predator odour was (H_3,39_ = 6.8, P = 0.001).

#### Test at Day 5

**Figure 4:**
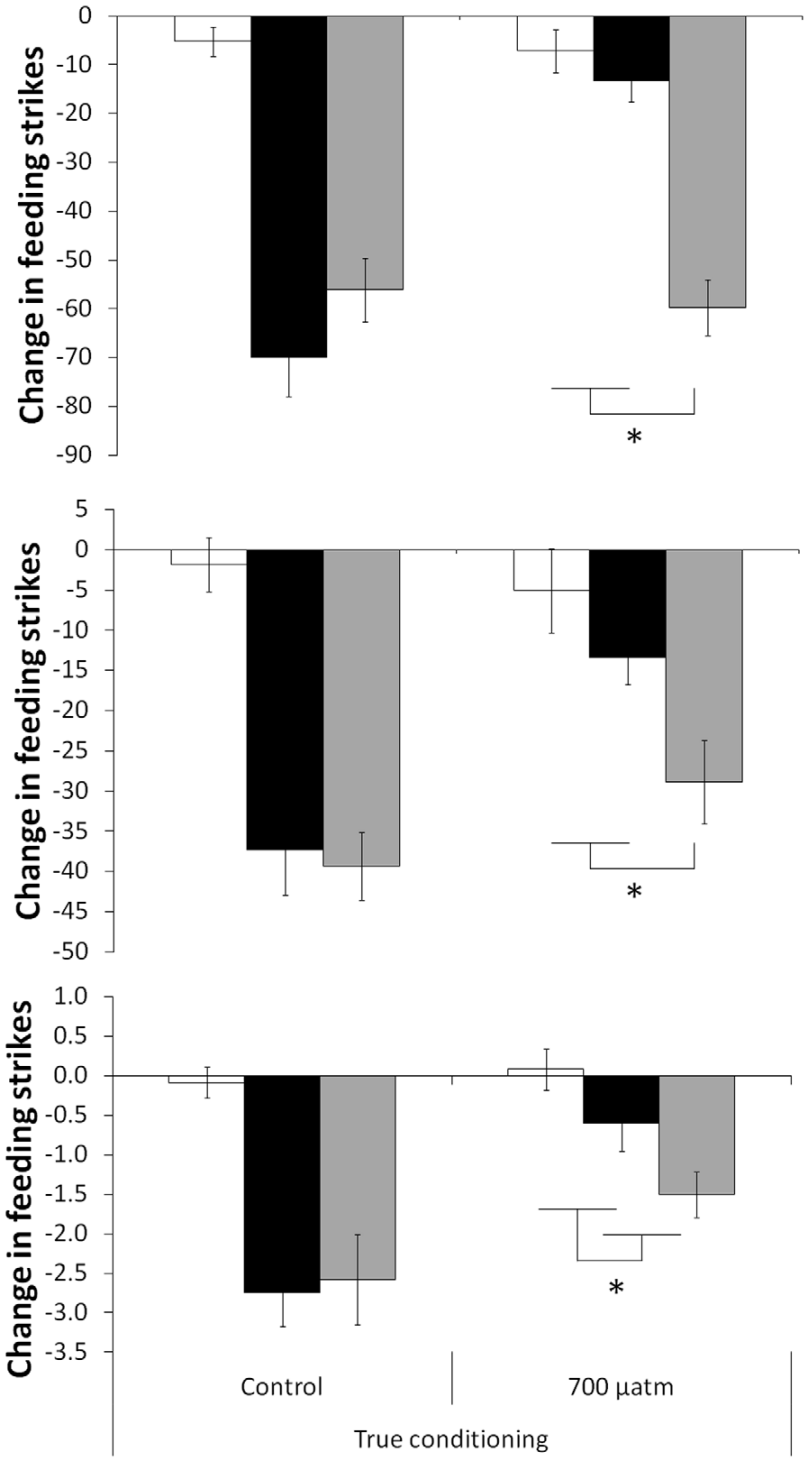
Mean change in number of feeding strikes (top), line crosses (middle) and area use (bottom) from the pre-stimulus baseline for fish conditioned to recognize a predator and then exposed to water (empty bars), predator odour (solid bars) or injured conspecific cues (grey bars) after five days. Fish were either raised under current-level CO_2_ (control) or elevated CO_2_ (700 µatm) (N = 12−15/treatment).

After the effects of CO_2_ wore off, we still found that fish's antipredator responses were influenced by both CO_2_ and cue (2×2 MANOVA, Pillai's Trace: F_6,136_ = 6.0, P<0.001, Figure 4). CO_2_ did not affect the responses of fish to water (1-way MANOVA, Pillai's Trace: F_3,20_ = 0.2, P>0.8) or a general risk cue like injured conspecific cues (F_3,20_ = 1.5, P>0.25), but did affect the responses of fish to predator odour (F_3,23_ = 13.7, P>0.001). Post-hoc tests revealed that fish did not respond differently to water and predator odour.

## Discussion

Learning through conditioning with odour cues of injured conspecifics and through observational social learning are very different processes, even though they lead to the same results. In the first, information about risk is provided by a chemical cue, while in the second, the information is provided by a visual source. The results of our study demonstrate that exposure of naïve juvenile fish to elevated levels of CO_2_ impairs both these processes. If our treatments represent future oceanic conditions on coral reefs, then evidence suggests that new recruit fishes will have a much reduced ability to assess predation risk and will as a consequence have much lower survival.

Our first experiment showed that juvenile damselfish exposed to control levels of CO_2_ were able to learn to recognise the odour cue of a predator, but juveniles exposed to 850 µatm CO_2_ were not. Our last experiment demonstrated that these effects also held at lower CO_2_ concentration (700 µatm CO_2_). In addition, once the CO_2_ effect wore off, fish conditioned to recognize the predator in elevated CO_2_ conditions still did not respond to the predator odour, but were able to display strong antipredator responses to other risk cues, such as injured conspecific cues. This indicates that elevated CO_2_ conditions did prevent learning from occurring.

Our work suggests that there is some form of cognitive impairment of the fish exposed to elevated CO_2_. The findings of our second experiment showed that larvae exposed to high levels of CO_2_ did not acquire recognition of the predator through cultural learning, whereas the control larvae were able to learn through this mechanism. Recent research showed that exposure to elevated CO_2_ affects both olfactory [15], [27] auditory [40] and visual [41] senses and a diverse range of behavioural activities in larval [16], [17] and adult fishes [42]. Furthermore, Domenici *et al.* [43] provides compelling evidence that elevated CO_2_ directly affects brain function in larval fishes, because behavioural lateralization (the propensity for individuals to turn left or right) is impaired by elevated CO_2_. The accumulating experimental evidence indicates that impaired and altered behaviour following exposure to elevated CO_2_ is caused by a systemic effect at the neurological level. A new study by Nilsson et al. [44] has confirmed this prediction by demonstrating that ionic changes associated with acid-base regulation interfere with brain neurotransmitter function in fish exposed to elevated CO_2_. Therefore, the broad range of behavioural problems identified in larval and juvenile fishes exposed to elevated CO_2_, including the impaired learning ability demonstrated here, appear to be caused by the ionic changes that fish use to prevent acidosis when permanently exposed to high CO_2_. We encourage researchers examining other environmental stressors to consider systemic neurological effects rather than focussing their attention on impaired sensory perception.

Like many ocean acidification studies, our CO_2_ treatment was short-term; hence we need to consider whether the responses we observed were a result of stress related to our methodology. However, previous studies have shown that raising coral reef fishes in CO_2_ from hatching lead to similar alterations in antipredator behaviour as those observed after a 4-day exposure to CO_2_ [16]. Hence, if our results were mediated by stress responses related to CO_2_ exposure, it seems likely than these effects cannot be dealt with through ontogeny. A possible alternative approach to studying the effects of ocean acidification may be through raising generations of fish in increasing CO_2_ conditions. However, beyond the limitation due to the life-history of some species (pelagic larvae), laboratory conditions may relax the selection pressures needed to maintain responses to predators.

Although larval fishes currently experience relatively stable CO_2_ conditions that are in equilibrium with the atmosphere during their pelagic stage in the open ocean, they may experience significant diurnal fluctuations in pCO2 once settled to the reef, temporarily approaching the levels used in our high- CO_2_ treatment. An obvious question that arises is: why are the fish able to learn to recognize predators under current conditions with this fluctuating CO_2_? The answer likely lies in the temporal aspect of the exposure regime. Previous research shows that behavioural impairment only occurs after several days of exposure to high CO_2_ and that impairment is retained for several days after larvae are returned to low pCO_2_ conditions [16]. Therefore, short term fluctuations in CO_2_ do not appear to impair learning.

Some conservation research has focused on means of increasing the survival of captive-bred [45] or translocated [46] individuals when released in an environment where these 1individuals are totally naive to their predators. A number of training programs have been undertaken to mitigate the ‘naivete’ effects, including social learning and conditioned learning, with some success [47], due to the power and efficacy of learning mechanisms to improve survival. In our situation however, it appears that learning mechanisms may be disrupted by these environmental conditions, which may impact the ability of prey to respond better to predators. The co-existence of affected and non-affected individuals towards mid-century will likely provide a great source of selection towards the elimination of individuals displaying maladaptive behaviour, both in a predation and homing context [16], [27]. This lack of response to predators could result in profound effects on coral reef community composition. Damselfish are common prey items for piscivores, especially following larval settlement on the reefs, and learning about predators is a very important way to decrease predation-related mortality [37]. A lack of response by larvae may lead to an increase in consumptive effects, which will change the amount of energy transferred to upper trophic levels. However, more works needs to be done on the effects of CO_2_ on foraging behaviours to predict how ocean acidification will affect predator-prey dynamics and trophic interactions.

While organisms typically exhibit a broad range of responses (physiological, morphological, life historical etc) to allow them to cope with current environmental conditions, behaviour is the one type of response that allow individuals flexibility to adjust to a wide range of conditions [48]. In the face of environmental change, behavioural responses typically occur first, as they occur faster, are more plastic and reversible than other forms of adaptations and allow the individual some control over its environment, by simply choosing the type of environment to live in. This crucial behavioural plasticity is often mediated through learning and limited or altered learning abilities may explain interspecific differences in the ability to respond appropriately to human-induced rapid environmental change [49]. Learning, that is, the ability to acquire new knowledge, skills, behaviour through experience, thereby changing the patterns of response to external stimuli, is an ability shared by virtually all animal species [20], [50]. Learning is crucial in allowing individuals to identify new suitable habitats or mates [51], food sources [20], new threats [52], and even adjust their behaviour and phenology in the face of environmental change [53], [54]. If CO_2_ exposure is altering the cognitive ability of species, by either preventing them from learning or by altering the interpretation of environmental cues, the ecological consequences of ocean acidification will be far reaching, and may impinge on any conservation efforts to mitigate the ecological effects of ocean acidification.
